# Anthropometric Indices in Adults: Which Is the Best Indicator to Identify Alanine Aminotransferase Levels?

**DOI:** 10.3390/ijerph13020226

**Published:** 2016-02-18

**Authors:** Shuang Chen, Xiaofan Guo, Shasha Yu, Ying Zhou, Zhao Li, Yingxian Sun

**Affiliations:** Department of Cardiology, the First Affiliated Hospital of China Medical University, Shenyang 110001, China; loscs@126.com (S.C.); guoxiaofan1986@foxmail.com (X.G.); yidasasa@foxmail.com (S.Y.); zhouying8111003@126.com (Y.Z.); meilichian@aliyun.com (Z.L.)

**Keywords:** alanine aminotransferase, body mass index, waist circumference, waist-to-height ratio, cross-sectional

## Abstract

*Background*: To evaluate the correlations between serum alanine aminotransferase (ALT) levels and anthropometric indices including body mass index (BMI), waist circumference (WC), hip circumference (HC), waist-to-height ratio (WHtR), waist-to-hip ratio (WHR), and a new body index, the A Body Shape Index (ABSI) in Chinese adults. *Methods*: A multicenter, cross-sectional study was conducted in rural areas of China in 2012–2013, and 11,331 adults were included in our final analysis. *Results*: BMI, WC, HC, WHtR, WHR and ABSI were significantly positively correlated with ALT levels. Spearman rank test showed that WHtR (*r* = 0.346 for men, *r* = 0.282 for women, both *p* < 0.001) had the highest correlation coefficient for ALT level, whereas ABSI showed the lowest, and the correlation coefficient of each measure was higher in men than that in women. Comparing the lowest with the highest quintile of each anthropometric measure, the multivariate logistic model presented that WHtR had the superiority of identifying the presence of elevated ALT (OR 4.38; 95% CI 3.15–6.08 for men, OR 4.29; 95% CI 2.91–6.33 for women, both *p* < 0.001), and the ABSI was the poorest predictor in men (OR 2.51; 95% CI 1.93–3.27, *p* < 0.001). No association was observed for ABSI in women. *Conclusions*: Our results indicated that BMI, WC, HC, WHtR and WHR were able to determine elevated ALT presence, while ABSI was not capable. WHtR and to some extent BMI were the best body indices, for predicting the ALT levels in this population. Nevertheless, the predictive ability of ABSI as a novel body index was not superior compared to established anthropometric indices.

## 1. Introduction

Among the liver enzymes, alanine aminotransferase (ALT) is used as a sensitive marker of a variety of hepatic damage [1,2]. Recent findings have shown that elevated ALT is associated with a range of health outcomes, such as metabolic disorders and cardiovascular diseases [3,4,5]. Nonalcoholic fatty liver disease (NAFLD), which is mainly due to liver fat accumulation, is considered as the main cause of elevated ALT in obese subjects [6,7,8]. Therefore, screening for ALT levels could contribute to identifying individuals at risk for these conditions. However, the confirmed diagnosis of NAFLD is the liver biopsy, and the blood collection of ALT level is invasive and could be impractical in large-scale, population-based epidemiological studies.

Measurements of anthropometric indices are non-invasive, inexpensive, and easily conducted in common healthy examination. If these anthropometric measurements showed a close correlation with serum ALT levels, they could be useful markers for predicting ALT levels. Until now, it has been reported that WC and BMI are positively associated with ALT levels [9,10,11,12]. Although BMI and WC are recommended by several guidelines to identify overweight and obesity [13,14], the discriminative accuracy of BMI and WC is not optimal. BMI is not sufficient to differentiate adipose tissue from lean body mass [15,16,17,18], and WC has been reported to be unclear to differentiate intra-abdominal from subcutaneous abdominal adiposity [19,20]. In fact, WC and WHR are the most commonly used markers of visceral adipose tissue, and they showed a positive relationship with increased risk of cardiovascular diseases [21]. However, it still remained controversial which anthropometric measure (BMI, WC, HC, WHR, or WHtR) was a more appropriate predictor of visceral adiposity and cardiovascular disease [22,23,24]. Indeed, a better body index could be designed on the basis of combining traditional measurements (e.g., weight, height, BMI, or WC). In 2012, Krakauer reported “A Body Shape Index” (ABSI), which is calculated by WC (m), BMI (kg/m^2^), and height (m). In that paper, the author showed a high ABSI was associated accumulation of excess abdominal adipose tissue [25].

The correlation between WC, BMI and elevated ALT levels has been extensively investigated, but there is insufficient evidence regarding the comparison of body indices for predicting the ALT levels. Especially in China, where the measurement of body indices and blood collection are not usually performed in the annual health examination, there have been few studies conducted among general population to date. Thus, the aim of this cross-sectional study is to investigate the correlations of serum ALT level with anthropometric measurements including BMI, WC, HC, WHtR, WHR and ABSI in a large-scale Chinese population.

## 2. Materials and Methods

### 2.1. Study Population

We conducted a cross-sectional study from July 2012 to August 2013 in rural areas of Liaoning Province, which is called Northeast China Rural Cardiovascular Health Study (NCRCHS). A representative sample aged ≥35 years was selected to describe the prevalence, incidence and natural history of cardiovascular risk factors. The study adopted a multi-stage, stratified randomly cluster-sampling scheme. In the first stage of sampling, 3 counties (Zhangwu, Dawa, and Liaoyang County) were randomly selected to represent south, east and north of Liaoning province. In the second stage, one town was randomly selected from each county (a total of 3 towns). In the third stage, 8–10 rural villages were randomly selected from each township. In total, 26 rural villages were finally included. All eligible permanent residents aged ≥35 years from each village were selected for participation (a total of 14,016 participants) and 11,956 individuals agreed and completed this cross-sectional study, making the response rate 85.3%. Approval for the NCRCHS was obtained from the Ethics Committee of China Medical University (Shenyang, China) (AF-SDP-07-1, 0-01). All participants provided written informed consent and all procedures were performed in accordance with the ethical standards. If the participants were illiterate, their proxies wrote the informed consents for them. In this study, we used data of baseline and only participants with complete data were included, making a final sample size of 11,331 (5243 men and 6088 women).

### 2.2. Data Collection

Data was collected during a single clinic visit by cardiologists and trained nurses using a standard questionnaire by face-to-face interview. Before the survey was performed, we invited all eligible investigators to attend the organized training. The training contents included the purpose of this study, how to administer the questionnaire, the standard method of measurement, the importance of standardization, and the study procedures. A strict test was evaluated after this training, and only those who scored perfectly on the test could become investigators. During data collection, our inspectors had further instructions and support.

Data on demographic characteristics, lifestyle risk factors, and medical history, were obtained by interview with a standardized questionnaire. There was a central steering committee with a subcommittee for quality control. Educational level was divided into primary school or below, middle school and high school or above. The smoking and alcohol consumption status were also surveyed. Smoking and alcohol status were assessed by two types of questions, “Have you ever smoked at least one cigarette per day for over six months/Have you ever taken alcohol at least twice a week for over a year?” and “Do you smoke/take alcohol now?” Respondents were defined as current smokers/drinkers (those who answered YES to both questions), former smokers/drinkers (those who answered YES to the first question and NO to the second one), and never smokers/drinkers (those who answered NO to both questions). Physical activity included occupational and leisure-time physical activity. Occupational and leisure-time physical activity were merged and regrouped into the following three categories: (1) low—subjects who reported light levels of both occupational and leisure-time physical activity; (2) moderate—subjects who reported moderate or high levels of either occupational or leisure-time physical activity; and (3) high—subjects who reported a moderate or high level of both occupational and leisure-time physical activity. Family income was classified as ≤5000, 5000–20,000 and >20,000 CNY/year.

### 2.3. Blood Pressure Measurements

According to American Heart Association protocol, blood pressure was measured three times in a sitting position at 2-min intervals after at least 5 min of rest in a quiet room with the use of an automatic electronic sphygmomanometer (HEM-741C; Omron, Tokyo, Japan). Two doctors checked the calibration of the Omron device using a standard mercury sphygmomanometer every month under the British Hypertension Society protocol [26]. The mean of three BP measurements was taken and used in all analyses.

### 2.4. Anthropometric Measurements

Standing height and weight were measured to the nearest 0.1 cm and 0.5 kg using a wall-mounted stadiometer and an automated balance. WC was measured at the minimum circumference between iliac crest and the rib cage in standing position at the end of normal expiration using a non-elastic tape, and HC at the level of the greater trochanters was measured using a flexible tape (to the nearest 0.1 cm). The body mass index (BMI) was calculated using the formula weight (kg)/height^2^ (m^2^). WHR was calculated by dividing WC by HC, and WHtR, by dividing WC by height. The ABSI was calculated using formula: ABSI = WC/(BMI^2/3^ × height^1/2^) [25].

### 2.5. Biochemical Measurements

Fasting (12 h overnight) blood samples were collected by venepuncture in EDTA tubes. Plasma was subsequently separated and frozen at −20 °C within 1 h for testing at a central, certified laboratory after collection. Fasting plasma glucose (FPG), plasma total cholesterol (TC), triglycerides (TG), low-density lipoprotein cholesterol (LDL-C), high-density lipoprotein cholesterol (HDL-C), serum uric acid (SUA), serum ALT and other biochemical parameters were analyzed enzymatically on an Olympus AU640 auto analyzer (Olympus, Kobe, Japan). All laboratory equipment was calibrated and blinded duplicate samples were used.

### 2.6. Definition of Elevated Serum ALT

Elevated serum ALT level was defined as ALT > 40 U/L [27].

### 2.7. Statistical Analysis

Continuous variables were expressed as mean values and standard deviation (SD), whereas categorical variables were described as frequencies and percentages. Continuous variables were compared between normal ALT and elevated ALT group by using Analysis of Variance (ANOVA) test. χ^2^-test analyses were used to examine associations between the categorical variables. Spearman’s correlation coefficients between anthropometric measurements (BMI, WC, HC, WHR, WHtR, and ABSI) and ALT levels were calculated. The correlation coefficient was applied for each sex. We used the area under the receiver-operating characteristic curve (AUC) and 95% confidence intervals (CIs) to assess the discriminatory power of each anthropometric measure to assess the risk for elevated ALT level. Quartiles of BMI, WHR and WHtR were created. For WC and HC, the quintiles were stratified by sex. Since ABSI was strongly correlated with age and sex [25], ABSI was stratified for four age groups (age 35–44, age 45–54, age 55–64 and age ≥ 65), after which ABSI quintiles were determined within each age group for males and females separately. Logistic regression was used to estimate the odds ratios (ORs) and 95% CIs for elevated ALT levels per quintile after adjustment for age, race, and lifestyle factors (smoking, drinking, family income, education, and physical activity). For all six anthropometric indices, the lowest quintile was set as reference. All statistical analyses were performed using SPSS version 19.0 software (SPSS Inc., Chicago, IL, USA), and *p* < 0.05 indicated statistical significance.

## 3. Results

### 3.1. Subject Characteristics

Comparisons of characteristics between men and women are shown in Table 1. A total of 11,331 adults (5243 men and 6088 women) aged ≥35 years were included in the study. The levels of ALT, uric acid, FPG, weight, height, HC, WC and WHR measurements for men were higher than the women’s, respectively. In contrast, the WHtR was higher in women. The BMI and ABSI showed no difference between sexes. Blood pressure (SBP and DBP) was higher in men, but TC and LDL-C levels were higher in women. There was no significant difference in TG and HDL-C between men and women.

### 3.2. Study Participants’ Characteristics According to ALT Level

Study population characteristics by sex and ALT level (ALT ≤ 40 and ALT > 40) are presented in Table 2. For both sexes, all of the anthropometric indices were larger in ALT > 40 than those in ALT ≤ 40. Both SBP and DBP were higher in the ALT > 40 group in men, but SBP showed no significant difference in women. Levels of ALT, uric acid, TC, TG, and LDL-C were higher, and HDL-C was lower in the ALT > 40 group for both sexes. In men, no statistical difference in FPG between the two groups was seen, compared to women who showed higher levels of FPG in the ALT > 40 group.

### 3.3. Correlation of Anthropometric Indices with Serum ALT Levels

Table 3 represents the correlation of serum ALT levels with anthropometric indices (BMI, WC, HC, WHR, WHtR, and ABSI). WHtR showed the highest Spearman correlation coefficient for ALT level (*r* = 0.346 for men and *r* = 0.282 for women, both *p* < 0.001), whereas ABSI showed the lowest coefficient (*r* = 0.101 for men and *r* = 0.093 for women, both *p* < 0.001) in both sexes. In addition, BMI and WC also showed relatively high coefficients (men: *r* = 0.330 for BMI and *r* = 0.310 for WC, women: *r* = 0.310 for BMI and WC, *p* < 0.001). Overall, the Spearman correlation coefficients were higher in men than in women.

### 3.4. The Logistic Regression Models for Elevated ALT and Each Anthropometric Index

The adjusted ORs for elevated ALT according to the quartiles of anthropometric indices are shown in Table 4. In general, the ORs of elevated ALT increased with increasing quartiles for all of the anthropometric measurements, after adjusting for age, race, smoking, drinking, education, physical activity, family income, history of medications, blood pressure, fasting plasma glucose, TC, TG, HDL, LDL, and uric acid. With the use of the ORs in the first quartile as a reference, considering the ORs of elevated ALT for the highest quartile of each anthropometric measurement, the WHtR was the best predictor of elevated ALT in both sexes (OR: 4.38, 95% CI: 3.15–6.08 in men; OR: 4.29, 95% CI: 2.91–6.33 in women, both *p* < 0.001). Although ABSI was the poorest predictor of elevated ALT in men (OR: 2.51, 95% CI: 1.93–3.21, *p* < 0.001), it showed no statistical significance for predicting elevated ALT in women. Compared to the ABSI, HC, WHR, and WC, the BMI was a better predictor for elevated ALT in both sexes (OR: 4.17, 95% CI: 3.15–5.54 in men; OR: 4.15, 95% CI: 2.78–6.19 in women, both *p* < 0.001).

### 3.5. The AUCs (and 95% CIs) of Anthropometric Measures for the Presence of Elevated ALT

**Figure 1:**
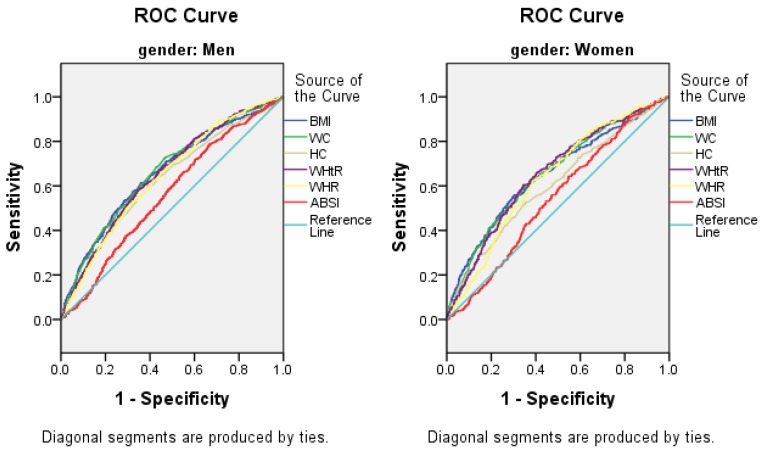
The discriminatory power of BMI, WC, HC, WHtR, WHR and ABSI in the prediction of elevated ALT. Area under the Receiver Operating Characteristic curve of BMI, WC, HC, WHtR, WHR and ABSI to identify subjects with elevated ALT according to sex. Abbreviations: BMI, body mass index; WC, waist circumference; HC, hip circumference; WHtR, waist-to-height ratio; WHR, waist-to-hip ratio; ABSI, a body shape index.

Elevated ALT was positively and significantly correlated to BMI, WC, HC, WHtR, WHR, and ABSI (Table 5; Figure 1). WHtR showed the highest AUCs for elevated ALT in both sexes (AUC: 0.664, 95% CI: 0.640–0.688 for men; AUC: 0.655, 95% CI: 0.622–0.688 for women), while the ABSI had the lowest AUCs for both genders (AUC: 0.561, 95% CI: 0.537–0.585 for men; AUC: 0.542, 95% CI: 0.511–0.573 for women).

## 4. Discussion

In the present study, we analyzed traditional and new anthropometric indices for their potential ability to predict serum ALT levels. Our results predicted that except for ABSI in women, all of the other anthropometric indices could identify elevated ALT levels after adjusting for age, race, smoking, drinking, education, physical activity, and family income. Moreover, the correlation coefficient of ALT levels was higher with WHtR than with BMI, WC, HC, WHR and ABSI. To the best of our knowledge, this is the first study regarding the relationship between different anthropometric measurements and ALT levels among large-scale general population in China, where physical examination and blood collection are not commonly performed in the annual health examination.

In agreement with previous studies [12,13,14], our results showed that WC had a positive correlation with ALT levels, and the correlation coefficient was relatively high. According to the modified NCEP-APT III (National Cholesterol Education Program-Adult Treatment Panel III) criteria, WC has proven to be a better surrogate for abdominal obesity [28]. Abdominal obesity causes excess release of free fatty acids and subsequent accumulation in hepatocytes, which may lead to fatty infiltration and decreased hepatocyte integrity [29,30]. Additionally, WC had greater potential for identifying subjects at increased risk of developing NAFLD [31].

Our results found that BMI had a strong relationship with elevated ALT levels in general population. The risk for elevated ALT levels tended to increase according to the increasing quintile of BMI. Similarly, Adams *et al.* showed among Australians, overweight (25 *≤* BMI < 30) and obese (BMI ≥ 30) subjects had an increased risk for the elevation of ALT. In another study conducted in American adults, it also revealed that the risk of elevated ALT activity was higher in overweight and obese groups [32]. Among British women aged 60 to 79 years, Lawlor *et al.* found a linear association of BMI with ALT concentration [33].

Consistent with our results, an earlier study had indicated that WHR was related to ALT levels [9]. The data from a Korean population showed that visceral fat accumulation tended to be associated with ALT levels [11], while WHtR was a stronger predictor of intra-abdominal fat than BMI and WC [34]. The results from these studies agree with our study, in which the WHtR was the most suitable for identifying elevated ALT, BMI and WC second, and the new anthropometric measurement (ABSI) showed the poorest capability to indicate the ALT levels.

Gender differences of the relationship between anthropometric indices and ALT levels existed in this study. All of the correlation coefficients between anthropometric indices and ALT levels in men were higher than those in women, and the gap between the 4th quartile and other quartiles in the stratified analysis was wider in men compared to women. A previous study also showed the OR for the association between WC and elevated ALT was higher in men than that in women [35]. Sex differences in the relation between ALT and anthropometric indices might be owing to sex hormones. It had been reported that sex hormones could affect the distribution of fat, and more intra-abdominal fat accumulation in men than in women [36]. In addition, a strong correlation was found between visceral fat accumulation and liver steatosis [37]. Furthermore, previous studies showed that the severity of fatty liver was positively associated with anthropometric indices including BMI and WC [38]. Another reason for the presence of sex differences could be due to differences in drinking status. In the present study, current smoking status was significantly more common in men (45.5%) than in women (2.9%).

In this study, anthropometric measurements (BMI, WC, HC, WHtR, WHR and ABSI) were positively correlated with ALT levels and the correlation coefficient of ALT levels was the highest with WHtR than with other indices. Furthermore, the OR of elevated ALT for the highest quartile of WHtR was the highest in both sexes. These results suggest that WHtR is a more effective tool for predicting ALT levels than other anthropometric indices, because the association between WHtR and ALT was strongest in both sexes.

Study strengths and limitations: The strengths of this study are its population-based design, large sample size, and the first comparison of the six anthropometric indices. Although some anthropometric measurements including BMI, WC and WHtR were reported separately to be positively associated with ALT levels, this is the first study to compare these indices (BMI, WC, HC, WHtR, WHR, and ABSI). Our study compared relationships between the ALT levels and body indices. These data are particularly important in the case of rural Chinese population who has relatively poor living standard and health resources. The advantages of using anthropometric measurements are inexpensive and easily conducted for annual health examination. Several limitations in this study need to be acknowledged. First, because of its cross-sectional design, we were unable to determine whether or not there was a causal association. Thus, the obtained associations in this study should be considered with caution. Second, despite extensive adjustment has been done in our study, we did not measure hepatitis B or C antibody, or fatty liver, which are known to be related to an elevated ALT. Third, residual confounding from detailed history of medications existed in this study. Thus, the possibility still existed that unmeasured confounders may explain part of the association.

## 5. Conclusions

In summary, our present study reported that WHtR was most closely associated with ALT levels than other anthropometric indices. This study suggests that it is more useful to monitor WHtR than BMI, WC, HC, WHR and ABSI as a surrogate for ALT levels among general population. Because measurements of body indices are relatively inexpensive and simple in a clinical setting, using the most suitable anthropometric measurement to identify individuals at high risk of elevated ALT and hepatic diseases who may benefit from early intervention has important public health implications for better prevention, diagnosis, and treatment. However, further prospective investigations are necessary to understand the predictive ability of different anthropometric indices as elevated ALT risk indicator.

## Figures and Tables

**Table 1:** Baseline characteristics of the study subjects (*N* = 11,331).

Variables	Men	Women	*p* Value	Total
	(*n* = 5243)	(*n* = 6088)		(*N* = 11,331)
Age, year	54.4 ± 10.8	53.4 ± 10.3	<0.001 *	53.8 ± 10.6
Race (Han), %	4966 (94.7)	5779 (94.9)	0.324	10,745 (94.8)
**Clinical characteristics**				
Current smoking status, %	2995 (57.1)	1004 (16.5)	<0.001 *	3999 (35.3)
Current drinking status, %	2385 (45.5)	176 (2.9)	<0.001 *	2561 (22.6)
Education, %			<0.001 *	
Primary school or below	2191 (41.8)	3456 (56.8)		5647 (49.8)
Middle school	2454 (46.8)	2162 (35.5)		4616 (40.7)
High school or above	598 (11.4)	470 (7.7)		1068 (9.4)
Physical activity, %			<0.001 *	
Low	1180 (22.5)	2188 (35.9)		3368 (29.7)
Moderate	3775 (72.0)	3555 (58.4)		7330 (64.7)
High	288 (5.5)	345 (5.7)		633 (5.6)
Family income, CNY/year, %			0.024 ^#^	
≤5000	696 (13.3)	707 (11.6)		1403 (12.4)
5000–20,000	2819 (53.8)	3366 (55.3)		6185 (54.6)
>20,000	1728 (33.0)	2015 (33.1)		3743 (33.0)
SBP, mm·Hg	143.6 ± 22.6	140.5 ± 24.0	<0.001 *	141.7 ± 23.4
DBP, mm·Hg	83.7 ± 11.8	80.5 ± 11.5	<0.001 *	82.0 ± 11.8
**Anthropometric indices**				
Weight, kg	68.6 ± 11.1	60.3 ± 10.1	<0.001 *	64.1 ± 11.4
Height, m	166.4 ± 6.3	155.6 ± 6.1	<0.001 *	160.6 ± 8.2
HC, cm	96.2 ± 7.1	95.4 ± 7.5	<0.001 *	95.8 ± 7.3
WC, cm	83.7 ± 9.7	81.2 ± 9.7	<0.001 *	82.4 ± 9.8
BMI, kg/m^2^	24.7 ± 3.5	24.9 ± 3.8	0.059	24.8 ± 3.7
WHtR	0.50 ± 0.06	0.52 ± 0.06	<0.001 *	0.51 ± 0.06
WHR	0.87 ± 0.08	0.85 ± 0.07	<0.001 *	0.86 ± 0.08
ABSI	0.0766 ± 0.0048	0.0767 ± 0.0055	0.525	0.0767 ± 0.0052
**Laboratory data**				
TC, mmol/L	5.2 ± 1.0	5.3 ± 1.1	<0.001 *	5.2 ± 1.1
TG, mmol/L	1.7 ± 1.6	1.6 ± 1.3	0.185	1.6 ± 1.5
HDL-C, mmol/L	1.4 ± 0.4	1.4 ± 0.3	0.722	1.4 ± 0.4
LDL-C, mmol/L	2.9 ± 0.8	3.0 ± 0.8	<0.001 *	2.9 ± 0.8
FPG, mmol/L	6.0 ± 1.7	5.9 ± 1.6	0.004 ^#^	5.9 ± 1.6
Serum uric acid, umol/L	333.7 ± 83.5	255.7 ± 67.7	<0.001 *	291.8 ± 84.4
ALT, U/L	25.6 ± 22.4	19.7 ± 13.0	<0.001 *	22.4 ± 18.2

Notes: Data are expressed as the mean ± SD or as *n* (%).Abbreviations: CNY, China Yuan (1 CNY = 0.161 USD); SBP, systolic blood pressure; DBP, diastolic blood pressure; HC, hip circumference; WC, waist circumference; BMI, body mass index; WHtR, waist-to-height ratio; WHR, waist-to-hip ratio; ABSI, a body shape index; TC, total cholesterol; TG, triacylglycerol; HDL-C, high-density lipoprotein cholesterol; LDL-C, low-density lipoprotein cholesterol; FPG, fasting plasma glucose; ALT, alanine aminotransferase. * *p* < 0.001; **^#^** *p* < 0.05.

**Table 2:** Characteristics of subjects according to the serum levels of ALT (*N* = 11,331).

Variables	Men (*n* = 5243)		*p* Value	Women (*n* = 6088)		*p* Value
	ALT *≤* 40	ALT > 40		ALT *≤* 40	ALT > 40	
	(*n* = 3375)	(*n* = 1432)		(*n* = 3375)	(*n* = 1432)	
Age, year	54.9 ± 10.8	49.7 ± 9.4	<0.001 *	54.4 ± 10.4	53.3 ± 9.0	0.936
Race (Han), %	4443 (94.7)	523 (95.1)	0.763	5505 (94.9)	274 (95.8)	0.581
**Clinical characteristics**						
Current smoking status, %	2726 (58.1)	269 (48.9)	<0.001 *	957 (16.5)	47 (16.4)	0.528
Current drinking status, %	2116 (45.1)	269 (48.9)	0.094	167 (2.9)	9 (3.1)	0.718
Education, %			<0.001 *			0.111
Primary school or below	200 (42.8)	182 (33.1)		3277 (56.5)	179 (62.6)	
Middle school	2162 (46.1)	292 (53.1)		2072 (35.7)	90 (31.5)	
High school or above	522 (11.1)	76 (13.8)		453 (7.8)	17 (5.9)	
Physical activity, %			0.580			0.049 ^#^
Low	1061 (22.6)	119 (21.6)		2066 (35.6)	122 (42.7)	
Moderate	3370 (71.8)	405 (73.6)		3407 (58.7)	148 (51.7)	
High	262 (5.6)	26 (4.7)		329 (5.7)	16 (5.6)	
Family income, CNY/year, %			<0.001 *			0.715
≤5000	654 (13.9)	42 (7.6)		677 (11.7)	30 (10.5)	
5000–20,000	2548 (54.3)	271 (49.3)		3210 (55.3)	156 (54.5)	
>20,000	1491 (31.8)	237 (43.1)		1915 (33.0)	100 (35.0)	
SBP, mm·Hg	143.4 ± 22.8	145.5 ± 20.3	0.021 ^#^	139.9 ± 24.0	142.6 ± 24.2	0.063
DBP, mm·Hg	83.8 ± 11.7	87.5 ± 11.9	<0.001 *	80.4 ± 11.5	82.6 ± 11.4	0.002 ^#^
**Anthropometric indices**						
Weight, kg	67.9 ± 10.7	74.5 ± 12.7	<0.001 *	60.0 ± 9.9	65.6 ± 12.1	<0.001 *
Height, m	166.3 ± 6.4	167.5 ± 6.1	<0.001 *	155.6 ± 6.1	155.9 ± 6.0	0.455
HC, cm	95.9 ± 7.0	98.7 ± 7.2	<0.001 *	95.3 ± 7.4	98.1 ± 7.9	<0.001 *
WC, cm	83.2 ± 9.5	88.8 ± 9.8	<0.001 *	81.0 ± 9.6	86.4 ± 10.1	<0.001 *
BMI, kg/m^2^	24.5 ± 3.4	26.5 ± 4.0	<0.001 *	24.7 ± 3.7	26.9 ± 4.4	<0.001 *
WHtR	0.50 ± 0.06	0.53 ± 0.06	<0.001 *	0.52 ± 0.06	0.55 ± 0.06	<0.001 *
WHR	0.87 ± 0.08	0.90 ± 0.06	<0.001 *	0.85 ± 0.08	0.88 ± 0.06	<0.001 *
ABSI	0.0765 ± 0.0049	0.0773 ± 0.0043	<0.001 *	0.0767 ± 0.0056	0.0772 ± 0.0045	<0.001 *
**Laboratory data**						
TC, mmol/L	5.1 ± 1.0	5.4 ± 1.2	<0.001 *	5.3 ± 1.1	5.8 ± 1.4	<0.001 *
TG, mmol/L	1.6 ± 1.5	2.4 ± 2.2	<0.001 *	1.6 ± 1.3	2.2 ± 1.9	<0.001 *
HDL-C, mmol/L	1.4 ± 0.4	1.4 ± 0.3	0.002 ^#^	1.4 ± 0.3	1.3 ± 0.4	<0.001 *
LDL-C, mmol/L	2.9 ± 0.8	3.0 ± 0.9	<0.001 *	3.0 ± 0.8	3.3 ± 1.0	<0.001 *
FPG, mmol/L	5.9 ± 1.7	6.1 ± 1.5	0.118	5.8 ± 1.6	6.2 ± 1.6	0.001 ^#^
Serum uric acid, umol/L	329.7 ± 81.2	368.4 ± 94.1	<0.001 *	254.3 ± 66.8	283.3 ± 78.2	<0.001 *
ALT, U/L	20.7 ± 7.6	67.2 ± 48.5	<0.001 *	17.6 ± 6.9	61.6 ± 28.6	0.036 ^#^

Notes: Data are expressed as the mean ± SD or as *n* (%).Abbreviations: CNY, China Yuan (1 CNY = 0.161 USD); SBP, systolic blood pressure; DBP, diastolic blood pressure; HC, hip circumference; WC, waist circumference; BMI, body mass index; WHtR, waist-to-height ratio; WHR, waist-to-hip ratio; ABSI, a body shape index; TC, total cholesterol; TG, triacylglycerol; HDL-C, high-density lipoprotein cholesterol; LDL-C, low-density lipoprotein cholesterol; FPG, fasting plasma glucose; ALT, alanine aminotransferase. * *p* < 0.001; **^#^** *p* < 0.05.

**Table 3:** Correlations of anthropometric indices (BMI, WC, HC, WHtR, WHR and ABSI) ^a^ with ALT.

Anthropometric Measurements	Men (*n* = 5243)		Women (*n* = 6088)	
	Coefficient (*r*)	*p* Value	Coefficient (*r*)	*p* Value
BMI, kg/m^2^	0.330	<0.001 *	0.269	<0.001 *
WC, cm	0.310	<0.001 *	0.269	<0.001 *
HC, cm	0.276	<0.001 *	0.198	<0.001 *
WHtR	0.346	<0.001 *	0.282	<0.001 *
WHR	0.253	<0.001 *	0.236	<0.001 *
ABSI	0.101	<0.001 *	0.093	<0.001 *

Notes: Abbreviations: BMI, body mass index; WC, waist circumference; HC, hip circumference; WHtR, waist-to-height ratio; WHR, waist-to-hip ratio; ABSI, a body shape index. ^a^ Independent variable for all models. * *p* < 0.001.

**Table 4:** Odd ratio and 95% confidence intervals for the risk of elevated ALT across quintiles of anthropometric measurements.

**Quintile (Men)**	**BMI**	**WC**	**HC**	**WHtR**	**WHR**	**ABSI**
**1 (Reference)**	**1**	**1**	**1**	**1**	**1**	**1**
2	1.79 (1.34, 2.40) *	1.09 (0.67, 1.77)	1.51 (1.14, 2.01) ^#^	1.82 (1.27, 2.61) ^#^	1.27 (0.92, 1.76)	1.15 (0.85, 1.56)
3	2.69 (2.03, 3.57) *	2.01 (1.31, 3.08) ^#^	1.94 (1.47, 2.56) *	2.53 (1.79, 3.58) *	2.06 (1.52, 2.79) *	1.61 (1.21, 2.13) ^#^
4	4.17 (3.15, 5.54) *	3.69 (2.47, 5.53) *	2.72 (2.06, 3.58) *	4.38 (3.15, 6.08) *	3.40 (2.54, 4.53) *	2.51 (1.93, 3.27) *
**Quintile (Women)**	**BMI**	**WC**	**HC**	**WHtR**	**WHR**	**ABSI**
**1 (Reference)**	**1**	**1**	**1**	**1**	**1**	**1**
2	2.08 (1.38, 3.16) ^#^	1.16 (0.83, 1.62)	1.19 (0.81, 1.75)	1.58 (1.02, 2.45) ^#^	1.06 (0.68, 1.63)	1.26 (0.87, 1.81)
3	3.47 (2.34, 5.14) *	2.33 (1.74, 3.11) *	1.46 (1.03, 2.08) ^#^	2.17 (1.43, 3.30) *	1.55 (1.04, 2.32) ^#^	1.78 (1.26, 2.53) ^#^
4	4.15 (2.78, 6.19) *	3.66 (2.77, 4.83) *	2.32 (1.64, 3.27) *	4.29 (2.91, 6.33) *	3.39 (2.38, 4.85) *	1.41 (0.96, 2.06)

Notes: The between cut points are 22.2, 24.6, and 27.0 for BMI; 77.0, 83.0, and 90.0 for WC (men); 75.0, 81.0, and 87.0 for WC (women); 2.81, 3.55, and 92.0, 96.0, and 100.2 for HC (men); 91.0, 95.0, and 100.0 for HC (women); 0.47, 0.51, and 0.55 for WHtR; 0.81, 0.86, and 0.91 for WHR; 0.0736, 0.0764, and 0.0794 for ABSI (men); 0.0732, 0.0764, and 0.0798 for ABSI (women). Abbreviations: BMI, body mass index; WC, waist circumference; HC, hip circumference; WHtR, waist-to-height ratio; WHR, waist-to-hip ratio; ABSI, a body shape index. ^a^ Adjusted for age, race, smoking, drinking, education, physical activity, family income, history of medications, blood pressure, fasting plasma glucose, TC, TG, HDL, LDL, and uric acid. * *p* < 0.001, **^#^** *p* < 0.05.

**Table 5:** Area under the Receiver Operating Characteristic curve (AUC) of each anthropometric measure for the presence of elevated ALT in both genders.

Anthropometric Measurements	Men (*n* = 5243)		Women (*n* = 6088)	
	AUC	95% CI	AUC	95% CI
BMI, kg/m^2^	0.658	0.633–0.683	0.651	0.616–0.685
WC, cm	0.651	0.627–0.674	0.653	0.621–0.685
HC, cm	0.626	0.601–0.651	0.602	0.568–0.637
WHtR	0.664	0.640–0.688	0.655	0.622–0.688
WHR	0.641	0.618–0.665	0.635	0.604–0.665
ABSI	0.561	0.537–0.585	0.542	0.511–0.573

Notes: BMI, body mass index; WC, waist circumference; HC, hip circumference; WHtR, waist-to-height ratio; WHR, waist-to-hip ratio; ABSI, a body shape index.

## Abbreviations

The following abbreviations are used in this manuscript:

SBP: systolic blood pressure
DBP: diastolic blood pressure
WC: waist circumference
BMI: body mass index
HC: hip circumference
WHtR: waist-to-height ratio
WHR: waist-to-hip ratio
ABSI: A Body Shape Index
TC: total cholesterol
TG: triacylglycerol
HDL-C: high-density lipoprotein cholesterol
LDL-C: low-density lipoprotein cholesterol
FPG: fasting plasma glucose
ALT: alanine aminotransferase
NAFLD: nonalcoholic fatty liver disease
OR: odds ratio
CI: confidence interval
